# Regional variations in the process of care for patients undergoing percutaneous coronary intervention in Japan

**DOI:** 10.1016/j.lanwpc.2022.100425

**Published:** 2022-03-15

**Authors:** Satoshi Shoji, Kyohei Yamaji, Alexander T. Sandhu, Nobuhiro Ikemura, Yasuyuki Shiraishi, Taku Inohara, Paul A. Heidenreich, Tetsuya Amano, Yuji Ikari, Shun Kohsaka

**Affiliations:** a Department of Cardiology, Hino Municipal Hospital, Tokyo, Japan; b Department of Cardiology, Keio University School of Medicine, 35 Shinanomachi, Shinjuku-ku, Tokyo 160-8582, Japan; c Department of Cardiovascular Medicine, Kyoto University Graduate School of Medicine, Kyoto, Japan; d Department of Medicine, Division of Cardiovascular Medicine, Stanford, CA, USA; e Medical Service, Veterans Affairs Palo Alto Health Care System, Palo Alto, CA, USA; f Department of Cardiology, Aichi Medical University, Aichi, Japan; g Department of Cardiology, Tokai University School of Medicine, Kanagawa, Japan

**Keywords:** Quality metrics, Regional variations, Percutaneous coronary intervention, Preprocedural testing, Coronary computed tomography angiography, Fractional flow reserve

## Abstract

**Background:**

Measuring the quality of care has been central for improving the outcomes of patients undergoing percutaneous coronary intervention (PCI). This study described the performance rates and regional variations in quality metrics for PCI using a representative national Japanese registry.

**Methods:**

Overall, 760,854 patients across 714 institutions (2016–2018) were analysed. Quality metrics included preprocedural antiplatelet therapy use, door-to-balloon time ≤90 min for ST-elevation myocardial infarction, transradial approach, and preprocedural noninvasive stress testing for elective cases in 47 Japanese prefectures. Coronary computed tomography angiography (CCTA) and fractional flow reserve (FFR) were also evaluated. Factors associated with preprocedural testing rates were evaluated using multivariable linear regression.

**Findings:**

Rates of preprocedural antiplatelet therapy use were high with low variations (96·4% [94·7–97·2%]), but there was still substantial room for improvement in the rates of door-to-balloon time (74·7% [71·2–78·9%]) and transradial approach use (70·9% [65·1–73·4%]). Rates of preprocedural noninvasive stress testing were low with substantial variation (36·6% [27·1–49·7%]). Additionally, we found substantial variations in CCTA (50·0% [39·5–55·1%]) and FFR measurement (15·7% [113·–18·3%]) rates. The number of scintigraphy scanners/ prefecture was associated with the performance of noninvasive stress testing (13·4% [95% CI, 2·45–24·4%] increase for every 1/100,000 population increase in scanners).

**Interpretations:**

We observed substantial regional variation in the use of preprocedural testing, and its performance was directly related to nuclear-scanner availability. These findings suggest the need for targeted efforts in improving testing rates, whether by optimising resource allocation or additional education or feedback mechanisms.

**Funding:**

This study was funded by the Japan Society for the Promotion of Science (Grant Nos. 20H03915, 16H05215, 16KK0186, and 20K22883) and by the Ministry of Health, Labor and Welfare Grants-in-Aid for Scientific Research Program (Grant No. 21FA1015). The J-PCI registry is led and supported by the Japanese Association of Cardiovascular Intervention and Therapeutics.

**Research in context**

**Evidence before this study**

Percutaneous coronary intervention remains widely employed in the management of coronary artery disease (more than 400,000 cases/year in the US and 200,000 in Japan). Numerous studies have demonstrated that measuring the quality of care to identify regional variations has been central for improving the outcomes of patients undergoing percutaneous coronary intervention. Previous studies on regional variations in the management of coronary artery disease have focused on patients with acute coronary syndrome; however, no studies have evaluated variations in the management of elective cases, comprising most percutaneous coronary interventions currently performed, and are more amenable to implementation efforts promoting standardised recommendations from clinical practice guidelines. To our knowledge, our study is the first to describe the achievement rates of and variations in quality metrics for both acute and elective cases and preprocedural testing before elective percutaneous coronary intervention in 47 prefectures in Japan using a representative national Japanese registry (J-PCI).

**Added value of this study**

We identified that performance rates of preprocedural antiplatelet therapy use was high with low variation, but there was still substantial room for improvement in rates of door-to-balloon-time and transradial approach use. Moreover, the rate of preprocedural noninvasive stress testing was low with substantial variation. Further, substantial variations in the use of coronary computed tomography angiography and fractional flow reserve even existed. Decreased availability of scintigraphy scanners was associated with lower rates of preprocedural noninvasive stress testing. Computed tomography was universally available in Japan, and the performance of coronary computed angiography was not related to the availability of computed tomography.

**Implications of all the available evidence**

Considering the variations in management patterns across regions, further monitoring of process-of-care measures and establishment of incentive mechanisms are warranted to ensure continuous quality-of-care improvement.

Alt-text: Unlabelled box

## Introduction

Percutaneous coronary intervention (PCI) is widely employed in the management of coronary artery disease,^1^ with more than 200,000 PCI procedures being performed annually in Japan.^2^ Patients undergoing PCI remain at substantial risk for periprocedural complications, including procedure-related myocardial infarction and bleeding, despite technical refinements and the development of novel therapeutic agents.^3^ Therefore, delivery of evidence-based preprocedural evaluation and in-hospital management are essential to optimise patient outcomes and medical-resource utilisation.^4^, ^5^, ^6^, ^7^, ^8^, ^9^

Numerous studies have demonstrated that measuring the quality of care to identify regional variations has been central for improving the outcomes of patients undergoing PCI.^10^, ^11^, ^12^, ^13^, ^14^ The research on regional variations in the management of coronary artery disease have focused on patients with acute coronary syndrome (ACS).^15^, ^16^, ^17^ Few studies have evaluated variations in the management of elective cases, comprising most PCIs that are currently performed, and are more amenable to implementation efforts promoting standardised recommendations from clinical practice guidelines.^18^, ^19^, ^20^

The Japanese Percutaneous Coronary Intervention (J-PCI) registry was launched by the Japanese Association of Cardiovascular Intervention and Therapeutics (CVIT) as a procedure-based registration system for PCI in 2013.^21^ The J-PCI registry closely works with the American College of Cardiology-National Cardiovascular Data Registry in the development of its quality metrics,^2^ and four quality metrics [(1) preprocedural antiplatelet (AP) therapy use, (2) door-to-balloon (DTB) time for ST-elevation myocardial infarction (STEMI) (≤90 min), (3) transradial approach (TRA), and (4) preprocedural noninvasive stress testing for stable ischaemic heart disease] were developed in 2017.^22^^,^^23^ This provided us with the first opportunity to describe the achievement rates of and variations in quality metrics for both acute and elective cases.

Hence, this study aimed to describe the performance rates and variations in the four quality metrics for both acute and elective cases and preprocedural testing [coronary computed tomography angiography (CCTA) and fractional flow reserve (FFR)] for elective cases in 47 prefectures in Japan using a representative national Japanese registry.

## Methods

The data and materials used to conduct this research are available to researchers for the purposes of reproducing the results or replicating the procedure on request. The procedure needs to follow the Act on the Protection of Personal Information Law (as of May 2017) and the Ethical Guidelines for Medical and Health Research Involving Human Subjects (as of March 2015) in Japan.

### Data source

The J-PCI registry is an ongoing nationwide prospective multicenter registry that is sponsored by the CVIT. It is designed to collect data on patient characteristics, clinical presentations, and angiographic and procedural details for all patients undergoing PCI.^21^ The definitions of variables in the J-PCI registry are available online (http://www.cvit.jp/files/registry/j-pci-definition.pdf). As registration in the J-PCI registry is mandatory for board certification and renewal application under both systems, data completeness is high. The annual reports of the Japanese Registry of All Cardiac and Vascular Diseases revealed that 816,374 PCIs were performed over the study period; therefore, approximately 93.2% of all PCIs were recorded in the J-PCI registry.^21^

Each hospital has a data manager who is responsible for data collection and database entry. The CVIT holds an annual meeting to ensure appropriate data collection and conducts random audits (20 institutions annually) to assess the quality of abstracted data. The study protocol of the J-PCI registry was approved by the Institutional Review Board Committee of the Network for the Promotion of Clinical Studies, a nonprofit organisation affiliated with Osaka University Graduate School of Medicine (Approval number: CVIT-2017). The requirement for written informed consent was waived owing to the retrospective study design.

### Study population

**Figure 1:**
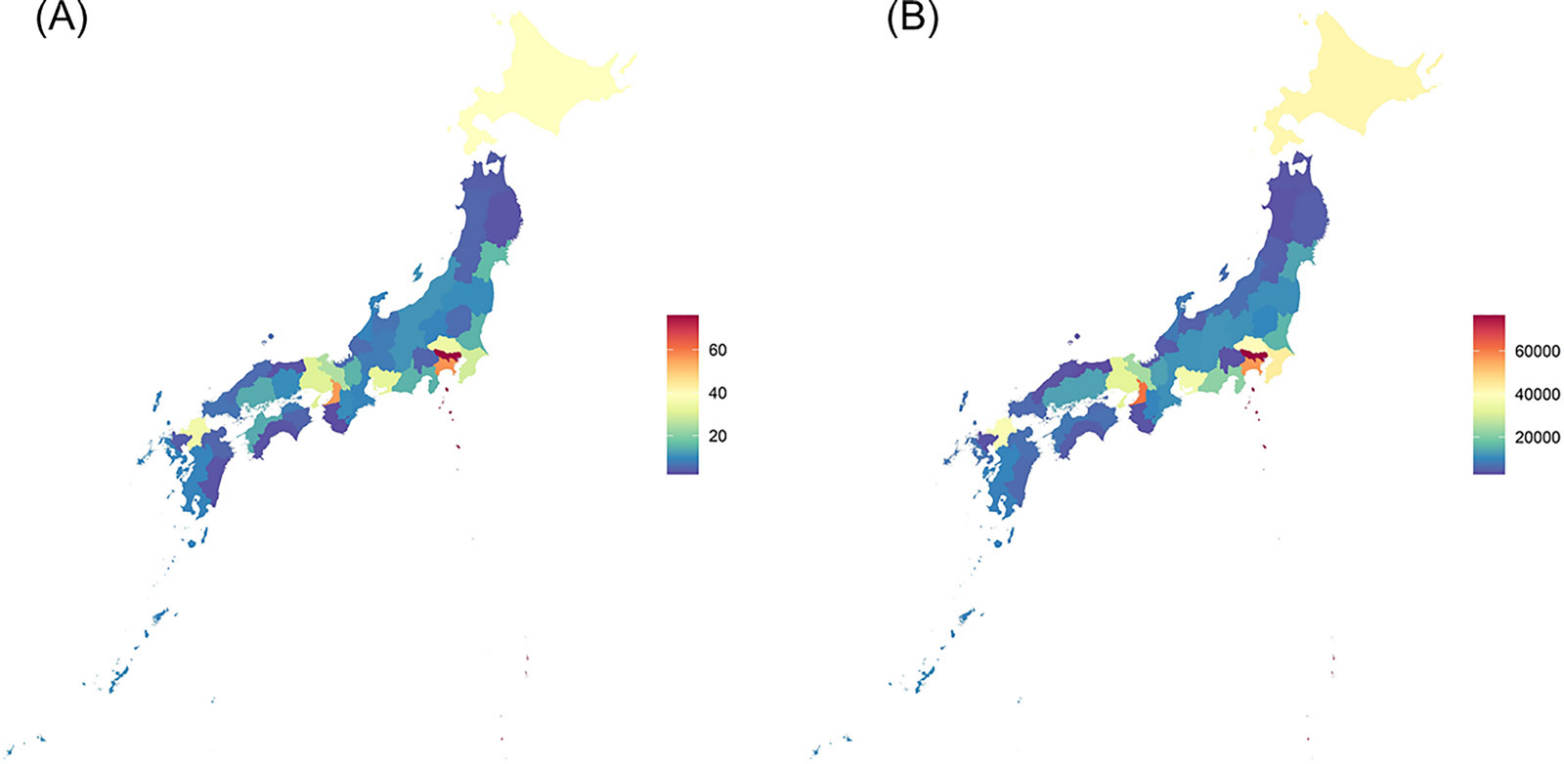
Distribution of (A) Institutions and (B) Patients in 47 Japan prefectures.

This study included 761,177 patients who underwent PCI from January 2016 to December 2018 at 714 institutions spread across 47 prefectures in Japan. The distribution of institutions according to prefectures is illustrated in Figure 1A. Patients with missing data on sex and age or those aged >99 or <21 years were excluded (*N* = 323, 0.004%).^21^ The final population comprised 760,854 patients. “Prefecture” was used as the unit of analysis in this study.

### Quality metrics and definition

The CVIT introduced quality metrics in 2017 to standardise the quality of PCIs,^22^^,^^23^ including (1) preprocedural AP therapy use, (2) DTB time for STEMI (≤90 min), (3) TRA, and (4) preprocedural noninvasive stress testing for stable ischaemic heart disease. The rationale for each quality measure is described below:

• The importance of aspirin administration before PCI is widely recognised.^22^^,^^24^ Consequently, AP therapy should be used before PCI in all cases.
• As a shorter DTB time is consistently associated with lower long-term mortality,^25^ clinical practice guidelines recommend a DTB time of ≤90 min, which is considered an important global quality metric in the management of STEMI.^22^^,^^26^
• Several randomised controlled trials have shown that the TRA reduces the incidence of adverse outcomes compared with transfemoral access.^27^^,^^28^ The latest European Society of Cardiology guidelines recommend TRA use as the first choice.^22^^,^^24^
• Before PCI for stable ischaemic heart disease, it is essential to assess the patient's individual risk (and to potentially exclude cases that are rarely appropriate for revascularisation [e.g., patients with anatomical stenosis without concurrent ischaemia]), and clinical practice guidelines recommend preprocedural noninvasive stress testing in most elective cases.^23^^,^^24^ Preprocedural noninvasive stress testing for this study included any of the following: stress electrocardiography (ECG), stress myocardial perfusion imaging (MPI), stress echocardiography, or stress magnetic resonance imaging (MRI).

In addition to the aforementioned quality measures, we evaluated rates of CCTA and FFR among patients with stable ischaemic heart disease. Although they are not noninvasive stress tests, both are important approaches for assessing patients with stable ischaemic heart disease. CCTA is considered both sensitive and specific for the diagnosis of coronary artery disease and is becoming an alternative to noninvasive stress tests given its comparatively high diagnostic accuracy.^29^^,^^30^ Additionally, FFR could theoretically serve as a substitute for noninvasive stress testing. Therefore, we evaluated implementation rates, variations, and temporal trends in CCTA and FFR among patients with stable ischaemic heart disease.

The DTB time was only evaluated among patients with STEMI, whereas preprocedural noninvasive stress testing, CCTA, and FFR were only evaluated for patients with stable ischaemic heart disease. The rates of preprocedural AP therapy and TRA use were evaluated among all patients with PCI. Proportions were based on the number of procedures, and better-quality performance was defined as a higher proportion for each variable.

### Definition of clinical variables

STEMI was characterised by ST-segment elevation in ≥2 contiguous leads (≥0.2 mV in the precordial lead at J point or ≥0.1 mV in the extremity lead), new left bundle branch block, or posterior myocardial infarction on 12-lead ECG with elevated levels of cardiac biomarker, which were identified as increased creatine kinase/creatine kinase myocardial band levels (two times higher than the normal values) or increased troponin levels (≥99th percentile).

### Availability of imaging devices

**Figure 2:**
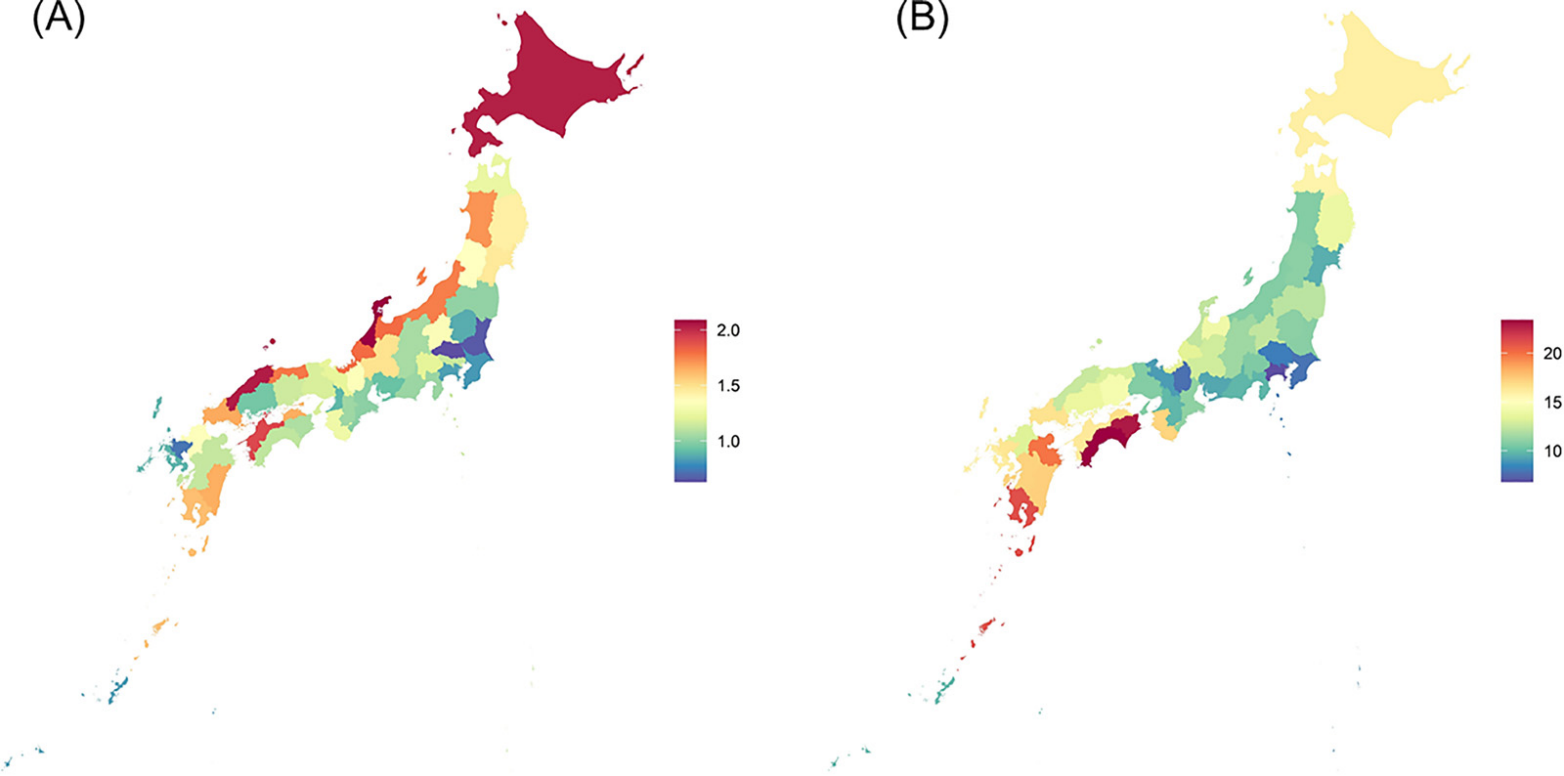
Number of (A) Scintigraphy and (B) Computed tomography scanners in 47 prefectures in Japan.

We determined the numbers of scintigraphy scanners (per 100,000 persons) and CT scanners (per 100,000 persons) in the 47 prefectures using data provided by the Japanese Ministry of Health, Labour, and Welfare (Figure 2).^31^

### Statistical analysis

Baseline characteristics and quality measures were compared among 47 Japanese prefectures. Normally distributed continuous variables are expressed as means with standard deviations, whereas other variables are presented as medians with 25th and 75th percentiles. Categorical variables are reported as proportions (%). Statistical comparisons between baseline characteristics were performed using an analysis of variance for normally distributed continuous variables and the Kruskal–Wallis test for non-normally distributed continuous variables. Pearson's chi-square test was employed for categorical variables.

We illustrated a scatter plot of the association between the imaging device availability (MPI and CCTA) and the implementation rate of the pre-PCI testing for the 47 prefectures. Factors associated with the implementation rate of each pre-PCI testing method were evaluated in each prefecture using multivariable linear regression. Age, sex, diabetes mellitus, renal failure, history of PCI, coronary artery bypass grafting, myocardial infarction, heart failure, haemoglobin level, dialysis, peripheral arterial disease, smoking status, and imaging device availability were incorporated as covariates.

Quality metrics were summarised according to a quarter of each year (2016–2018). The FFR data, which became newly available in 2017, were compared within the limited timeframe. The Cochran–Armitage test was used to evaluate temporal trends.

All variables had <3% missingness. Considering the small percentage of missing data, imputation was not applied. All statistical analyses were performed using R version 3.6.2 (R Foundation for Statistical Computing, Vienna, Austria) and SAS version 9.4 (SAS Institute, Cary, NC, USA). All *P*-values were two-sided, and *P-*value of <·05 was considered significant.

### Role of the funding source

The funder of the study had no role in study design, data collection, data analysis, data interpretation, or writing of the report. The corresponding authors had full access to all the data in the study and had final responsibility for the decision to submit for publication.

## Results

### Baseline characteristics

The mean age of the study population was 70·5 ± 11·1 years, and 23·8% were female. Overall, 77·7%, 67·2%, and 46·0% of the patients had hypertension, dyslipidemia, and diabetes mellitus, respectively. Furthermore, 46·5%, 22·8%, and 14·2% had previous history of PCI, myocardial infarction, and heart failure, respectively. Additionally, 19·3% of the patients had renal failure, whereas 7·1% were receiving haemodialysis (Table 1).

**Table 1:** Baseline patient characteristics in the studied dataset (J-PCI, from January 2016 to December 2018)

	Total
Clinical Variables	*N* = 760,854
Age, years, mean ± SD	70·5 ± 11·1
Female	180,704 (23·8%)
Diagnosis	
Acute setting	
STEMI	129,436 (17·0%)
NSTEMI	38162 (5·02%)
Unstable angina	114,956 (15·1%)
Elective setting	
Stable angina	273,274 (36·0%)
Old myocardial infarction	38,517 (5·07%)
Silent ischaemic heart disease	118861 (15·6%)
Staged PCI	33,392 (4·4%)
Previous PCI	348,795 (46·5%)
Previous CABG	27,442 (3·66%)
Previous myocardial infarction	170,320 (22·8%)
Diabetes mellitus	335,556 (46·0%)
Hypertension	566,147 (77·7%)
Dyslipidemia	489,792 (67·2%)
Current smoker	231,715 (31·8%)
Renal insufficiency	140,415 (19·3%)
On dialysis	51,658 (7·09%)
Chronic lung disease	18,166 (2·49%)
Peripheral artery disease	57,749 (7·92%)
Previous heart failure	105,557 (14·2%)
Cardiopulmonary arrest on arrival	13,826 (1·85%)
Cardiogenic shock within 24 h	24,418 (3·26%)
Acute heart failure within 24 h	30,649 (4·10%)
Haemoglobin, g/dL, mean (SD)	13.2 (2·05)
Creatinine, mg/dL, mean (SD)	1.39 (1·88)
**Lesion characteristics**	
Number of diseased vessels	
One-vessel disease	479,043 (63·0%)
Two-vessel disease	190,309 (25·0%)
Three-vessel disease	89,281 (11·7%)
Left main trunk	30,250 (3·98%)
Lesion location	
RCA	255,345 (33·6%)
LAD/left main	396,089 (52·1%)
LCX	189,332 (24·9%)
Bypass graft	3,622 (0·476%)
**Procedural details**	
Approach	
Transradial	524,148 (68·9%)
Transfemoral	197,517 (26·0%)
Others (e.g., brachial)	39,112 (5·14%)
Successful rate	739,695 (97·2%)
Intracoronary device used	
Thrombus aspiration*	90,632 (11·9%)
Distal protection	21,564 (2·83%)
Rotablator	29,071 (3·82%)
Drug-eluting stent	646,302 (84·9%)
Bare metal stent	9,326 (1·23%)

⁎ Confined to patients with STEMI. CABG, coronary artery bypass grafting; J-PCI, Japanese Percutaneous Coronary Intervention registry; LAD, left anterior descending; LCX, left circumflex; NSTEMI, non-ST-elevation myocardial infarction; PCI, percutaneous coronary intervention; RCA, right coronary artery; SD, standard deviation; STEMI, ST-elevation myocardial infarction.

From January 2016 to December 2018, 760,854 PCIs were recorded in the J-PCI registry. Of these, 62·9% were performed as elective cases. Throughout the study period, the number of PCIs was relatively stable; however, the number of PCIs for ACS slightly increased (90,857 cases in 2016; 94,996 in 2017; and 96,701 in 2018). Moreover, the number of patients undergoing elective PCIs remained stable during the study period (152,481 cases in 2016; 165,126 in 2017; and 160,693 in 2018). Patients’ distribution across prefectures is illustrated in Figure 1B.

### Variations in quality measures stratified by the 47 prefectures

**Figure 3:**
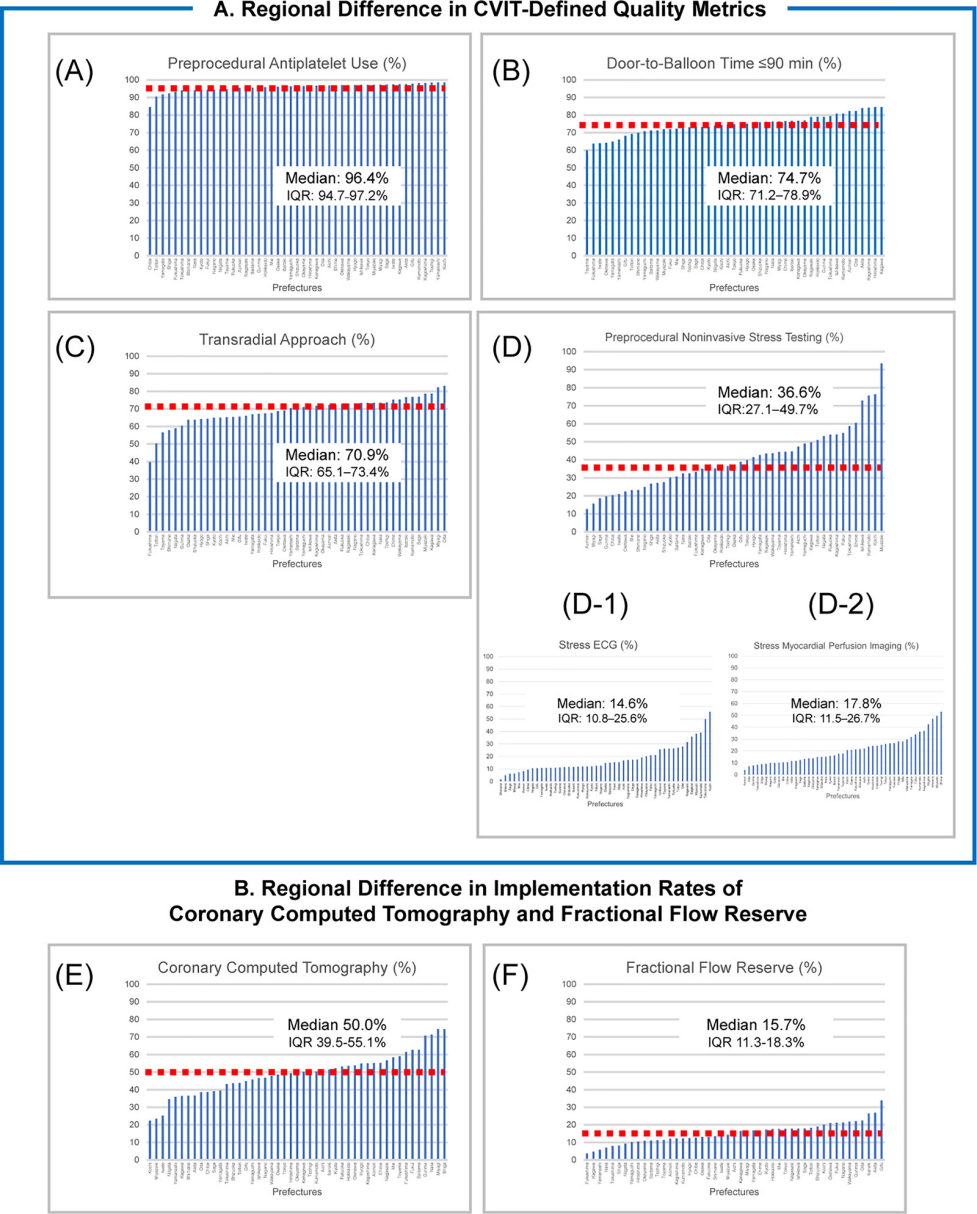
Performance rates of and variations in quality metrics and preprocedural testing stratified by 47 prefectures CVIT, Japanese association of cardiovascular intervention and therapeutics; ECG, electrocardiography.

The performance rates and variations in CVIT-defined quality metrics and preprocedural testing in the J-PCI registry stratified by the 47 prefectures are presented in Figure 3. The rates of preprocedural AP therapy use were high, with low variation (median, 96·4%; interquartile range [IQR], 94·7–97·2%; Figure 3A). However, performance rates for DTB time ≤90 min in patients with STEMI (median, 74·7%; IQR, 71·2%–78·9%; Figure 3B) and TRA use (median, 70·9%; IQR, 65·1–73·4%; Figure 3C) were relatively high with small variation, but there was still substantial room for improvement.

There was substantial variation in the use of preprocedural noninvasive stress testing (median, 36·6%; IQR, 27·1–49·7%; Figure 3D), particularly when divided based on the use of stress ECG (median, 14·6%; IQR, 10·8%–25·6%; Figure 3D-1) and stress MPI (median, 17·8%; IQR, 11·5–26·7%; Figure 3D-2). Fewer tests were performed with stress echocardiography (median, 0·25%; IQR, 0·09–0·83%) and stress MRI (median, 0·16%; IQR, 0·06–0·45%). Substantial variation in the use of CCTA (median, 50·0%; IQR, 39·5%–55·1%; Figure 3E) and FFR (median, 15·7%; IQR, 11·3–18·3%; Figure 3F) was also observed.

### Utilisation of Pre-PCI testing: association with availability of imaging devices

The association between the imaging device availability [the numbers of scintigraphy scanners (per 100,000 persons) and CT scanners (per 100,000 persons) in the 47 prefectures] and the implementation rate of the pre-PCI testing (MPI and CCTA per 47 prefectures) is shown in Supplemental Figure 1. The number of scintigraphy scanners in each prefecture was associated with MPI implementation (regression coefficient, 13·4% increase for every 1/100,000 population increase in scanners; 95% confidence interval [CI], 2·45 to 24·4%; *p* = 0·018). Histories of coronary artery bypass grafting and myocardial infarction were associated with lower rates of CCTA (regression coefficient, –4·4%; 95% CI, –8·77% to –0·08%; *p* = 0·046 and regression coefficient, –1·30%; 95% CI, –2·57% to –0·05%; *p* = 0·042, respectively). CT scanners were universally available throughout the country. The number of CT scanners was not associated with CCTA implementation (regression coefficient, –0·52% increase for every 1/100,000 population increase in scanners; 95% CI, –1·77% to 0·73%; *p =* 0·40).

### Temporal trends in quality measures

**Figure 4:**
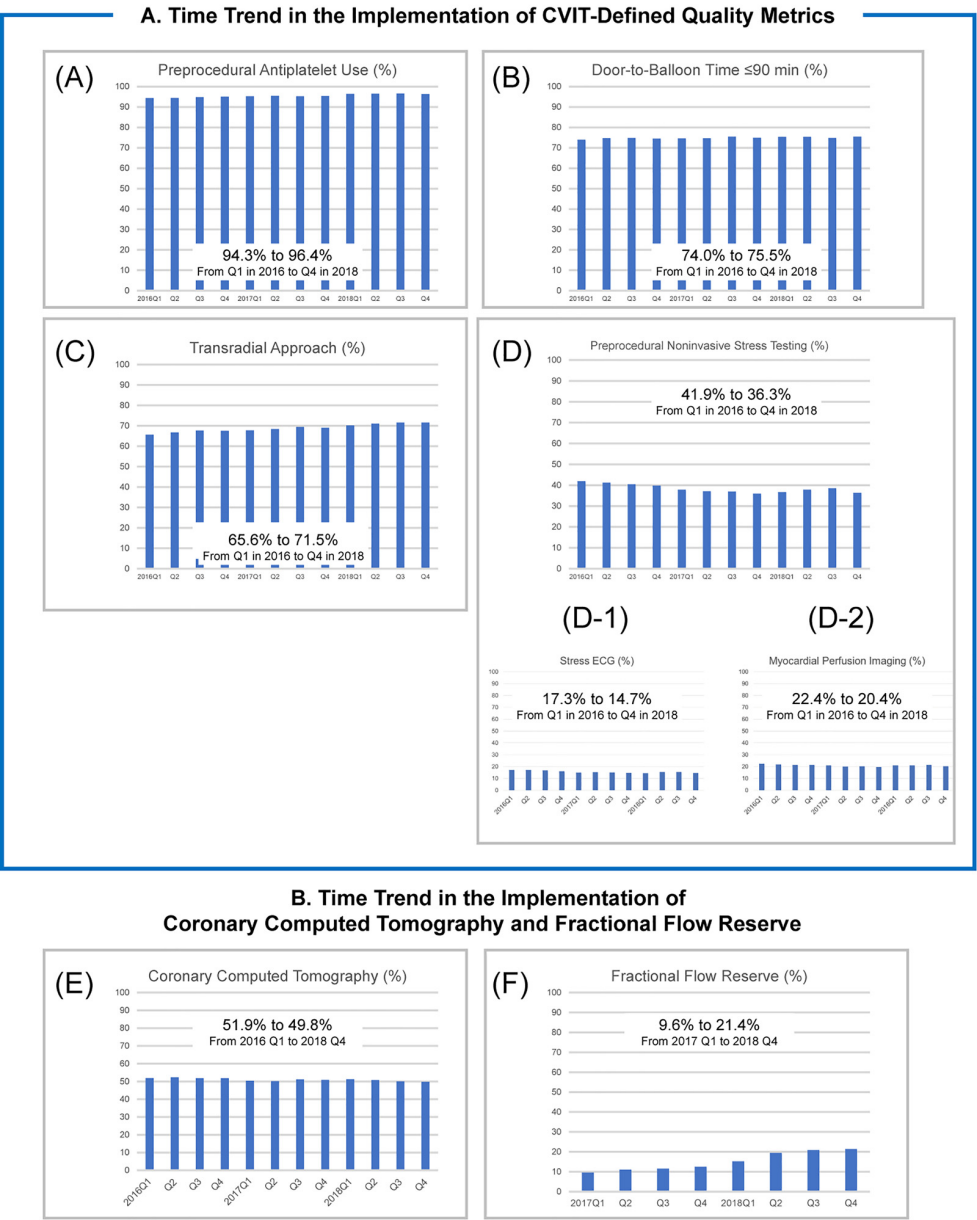
Trends in the performance rates of quality metrics and preprocedural testing CVIT, Japanese association of cardiovascular intervention and therapeutics; ECG, electrocardiography.

The overall rates of preprocedural AP therapy use, DTB time ≤90 min in patients with STEMI, and TRA use slightly improved during the study period (*P-*for-trend <0 ·001; Figure 4A–C). The use of preprocedural noninvasive stress testing (Q1 in 2016, 41·9%; Q4 in 2018, 36·3%) and CCTA (Q1 in 2016, 51·9%; Q4 in 2018, 49·8%) did not change over time, whereas the rates of FFR increased (Q1 in 2017, 9·6%; Q4 in 2018, 21·4%) (*P*-for-trend <0·001; Figure 4D–F).

## Discussion

In this study, we analysed a nationally representative database to provide contemporary data on real-world variations in quality metrics and preprocedural testing among patients undergoing PCI across Japan. Our analysis revealed the following: (1) When Japan was stratified into 47 prefectures, the rate of preprocedural AP therapy was high, with relatively low variation across prefectures, but there was still substantial room for improvement in the rates of DTB time ≤90 min in patients with STEMI (median, 74·7%; IQR, 71·2–78·9%) and TRA use (median, 70·9%; IQR, 65·1–73.4%). (2) The rates of preprocedural noninvasive stress testing are low, with substantial variation (median, 36·6%; IQR, 27·1–49·7%). (3) Substantial variations in the use of CCTA (median, 50·0%; IQR, 39·5–55·1%) and FFR (median, 15.7%; IQR, 11·3–18·3%) were also observed. The number of scintigraphy scanners in each prefecture was highly variable and associated with rates of preprocedural noninvasive stress testing; by contrast, CT scanners were universally available throughout the country, and their number was not associated with CCTA implementation. Our findings underscore the presence of gaps and variations in the preprocedural evaluation of stable ischaemic heart disease in Japan. Given that testing rates were strongly associated with availability, there is a possible necessity for optimising resource allocation to target areas with inadequate testing availability. Better understanding regarding other causes of regional variations in care may benefit the government's layout planning and reforms.

Quality metrics are utilised in quality improvement initiatives of various countries to continuously monitor and enhance the quality of care provided to patients undergoing PCI.^32^ Inohara et al. previously reported that the performance rates for DTB time ≤90 min in patients with STEMI and the use of preprocedural noninvasive stress testing in Japan were lower than those in the Unites States, whereas the use of TRA was substantially higher,^2^ and these trends were consistent with previous studies.^17^ To the best of our knowledge, this study is among the first to describe regional variations regarding real-world quality measure performance among a wide range of patients undergoing PCI (on both elective and urgent/emergent bases). Presenting regional variations in the use of quality metrics will help elucidate the extent to which these metrics are achieved by practicing physicians in daily clinical practice. Japan is a racially homogeneous nation and has universal health coverage, suggesting that there are limited factors leading to the observed regional differences. Our results provide a guide in terms of critical targets for improving the quality of care for PCI.^12^ Furthermore, Japan is one of the fastest ageing countries, and the number of Japanese adults aged ≥65 years constitutes nearly a quarter of the total population.^33^ The unique healthcare policies in Japan have led to outstanding population health and equity at low cost^34^; however, the burden of the ageing society has placed pressures on the appropriate use of healthcare resources and expenditures in recent years.^35^ In this context, CVIT introduced quality metrics in 2017 to standardise the quality of PCIs in the nation, and our findings may provide valuable insights to other countries.^36^ Understanding such regional variations has traditionally helped in identifying important quality gaps and has served to lay a foundation for future quality improvement initiatives across the country.

Preprocedural noninvasive stress testing, including stress ECG, stress MPI, stress echocardiography, and stress MRI, in patients undergoing initial evaluation for suspected stable ischaemic heart disease, remains the gold standard for both diagnosis and risk stratification in the United States.^37^ However, our study showed that CCTA was more frequently used than preprocedural noninvasive stress testing and that substantial variations in the use of functional testing and CCTA existed across the 47 Japanese prefectures. CCTA provides important prognostic information and has been increasingly used in clinical practice in the last 20 years worldwide.^38^^,^^39^ Several recent randomised controlled trials have shown that CCTA is associated with a reduced rate of myocardial infarction compared with functional stress testing, and clinical practice guidelines currently endorse the IIA recommendations for CCTA use when stress ECG testing is impractical or stress MPI testing is not evaluable.^40^, ^41^, ^42^ Nevertheless, recent studies have reported that CCTA is associated with increased rates of invasive procedures and high costs with a similar risk of all-cause mortality^38^^,^^43^, ^44^, ^45^; the common use of CCTA seen here should be interpreted with caution. Moreover, the use of scintigraphy was significantly associated with the availability of scintigraphy scanners, suggesting that access to scintigraphy strongly influences the choice of the initial testing strategy. This highlights the potential for improvement if policymakers optimise the availability of cardiac resource, which may enable physicians to select the initial testing strategy based on the pretest probability of ischaemia.

Notably, the use of FFR has dramatically increased since 2018. This phenomenon could be explained by the new reimbursement requirement for elective PCI by the Japanese Ministry of Health, Labour, and Welfare, which mandates preprocedural stress testing (including FFR) for preprocedural ischaemia evaluation. The relatively common use of FFR in Japan may also be related to the fact that anatomical information has traditionally been considered the most decisive factor in performing PCI, with CCTA being the most common preprocedural evaluation in Japan. Nonetheless, considering the invasiveness of FFR compared with that of other preprocedural stress testing modalities, further studies are necessary to confirm the superiority of FFR over preprocedural noninvasive stress testing.

In the United States, several PCI quality metrics are monitored by national registries, which periodically provide each institute with performance feedback for quality-of-care improvement. This includes efforts to reduce the number of elective PCIs classified as rarely appropriate. In Japan, a national PCI enrollment system was launched in 2013. The CVIT introduced quality metrics for Japanese citizens in 2017. Our study provides the first opportunity to describe the heterogeneity in performance which will enable poorly performing regions to learn from areas with better performance. As a foundation for quality improvement in Japan, we sought to better understand the quality of care and to provide opportunities for changing clinical practice by giving feedback to physicians and institutions.

### Limitations

Our study results should be interpreted in the context of some limitations and considerations. First, not all institutions in Japan participated in the J-PCI registry (93.2%). The remaining 6% would be patients who undergo PCI in hospitals not accredited by CVIT. Therefore, the performance rate of CVIT-defined quality metrics in hospitals not accredited by CVIT would be more likely to be lower than that in hospitals registered in the J-PCI. Second, the registration system of the J-PCI registry was only launched in 2013. Therefore, several variables were newly added during the study period. Consequently, some important variables, such as FFR, were compared within a limited timeframe. Third, since important clinical variables such as ethnicity or body mass index were not collected in the J-PCI registry, we could not adjust the multivariable models using these variables. Fourth, other potential measures can be considered for quality metrics, such as optimal medical therapy and anti-anginal therapy before revascularisation in patients with stable ischaemic heart disease (e.g., beta-blockers, nitrates, and statins). We will periodically update the quality metrics as new data and evidence become available. Finally, data on access to scintigraphy and CT equipment for each patient were not available; hence, we used the data on the total number of scintigraphy and CT scanners in each of the 47 prefectures.

## Conclusions

Using a representative Japanese registry, we identified that the performance rates of preprocedural AP therapy use was high with low variation, but there was still substantial room for improvement in the rates of DTB and TRA use. Moreover, the rate of preprocedural noninvasive stress testing was low, and there was substantial variation. There was also substantial variation in the use of CCTA and FFR before PCI. Lower availability of scintigraphy scanners was associated with lower rates of preprocedural noninvasive stress testing. The overall rates of preprocedural noninvasive stress testing and CCTA did not change over time, whereas the FFR rates increased. Considering the variations in management patterns across regions, further monitoring of process-of-care measures and establishment of incentive mechanisms are warranted to ensure continuous quality-of-care improvement.

### Author contributions

Dr. Kohsaka takes responsibility for the integrity of the data and the accuracy of the data analysis.

Concept and design: Shoji, Yamaji, Kohsaka.

Acquisition, analysis, or interpretation of data: Shoji, Yamaji, Inohara, Shiraishi, Ikemura, Kohsaka

Drafting of the manuscript: Shoji, Kohsaka.

Critical revision of the manuscript for important intellectual content: Sandhu, Heidenreich.

Statistical analysis: Yamaji.

Obtained funding: Shoji, Kohsaka.

Administrative, technical, or material support: Amano, Ikari.

Supervision: Heidenreich, Kohsaka

### Data sharing statement

The data and materials used to conduct this research are available to researchers for purposes of reproducing the results or replicating the procedure on request. The procedure does need to follow the Act on the Protection of Personal Information Law (as of May 2017) and the Ethical Guidelines for Medical and Health Research Involving Human Subjects (as of March 2015) in Japan.

*Editor note: The Lancet Group takes a neutral position with respect to territorial claims in published maps and institutional affiliations*.

## Declaration of interests

Kohsaka has received investigator-initiated grant funding from Daiichi-Sankyo and Bristol-Myers Squibb and has received personal fees from Bristol-Myers Squibb. All other authors have reported that they have no relationships relevant to the contents of this paper to disclose.

## Appendix Supplementary materials

Image, application 1
