# Supplementary material for: Regional variations in the process of care for patients undergoing percutaneous coronary intervention in Japan
Source: Lancet Reg Health West Pac. 2022 Mar 15;22:100425. doi: 10.1016/j.lanwpc.2022.100425 (PMC8928076; doi:10.1016/j.lanwpc.2022.100425)

**Supplemental Materials**

**Supplemental Figure 1. Association between the imaging device availability and the implementation rate of the pre-percutaneous coronary intervention testing per 47 prefectures.**

**(A) Association between the numbers of scintigraphy scanners (per 100,000 persons) and the implementation rate of scintigraphy for the 47 prefectures**


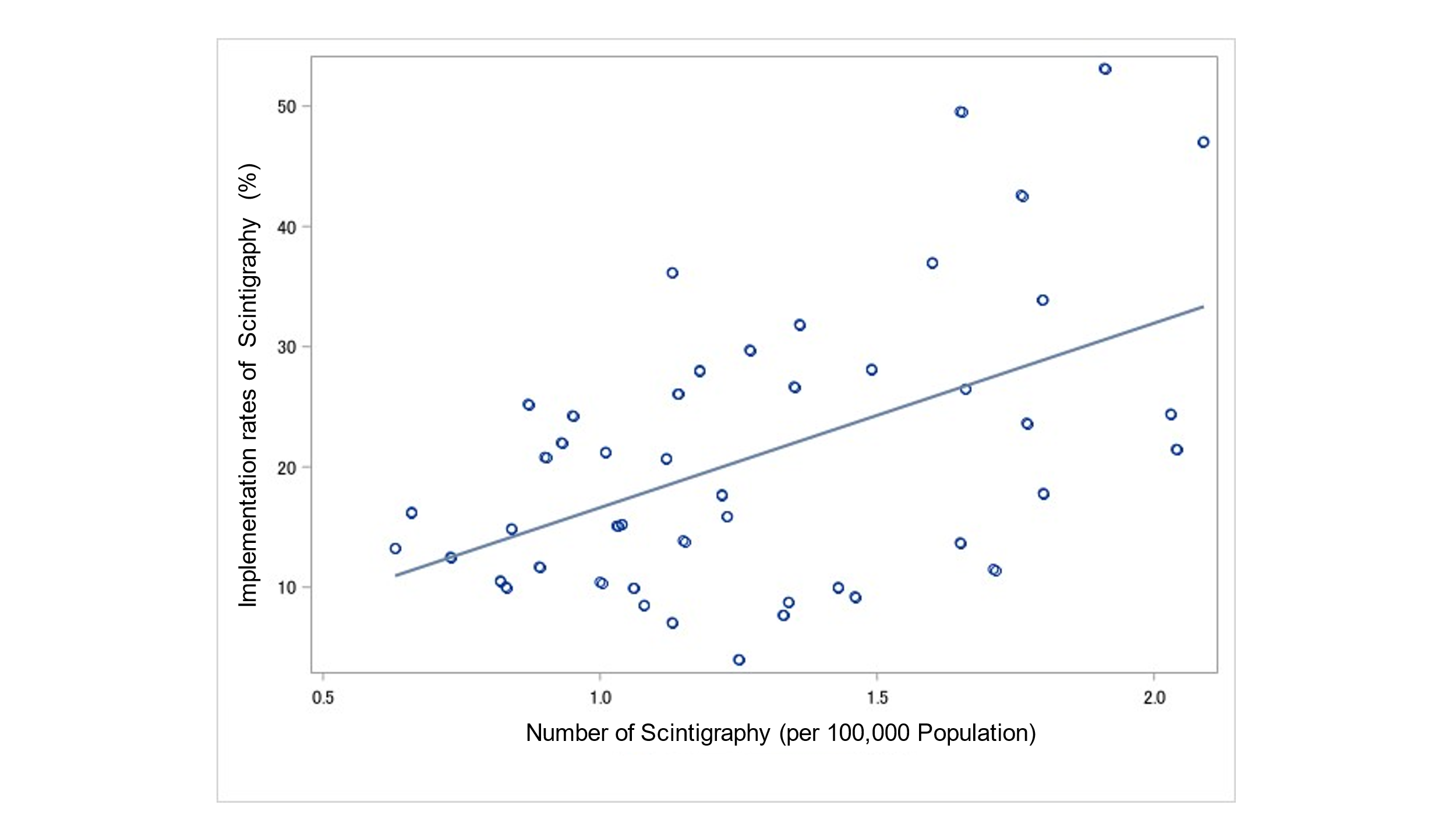


**(B) Association between the numbers of computed tomography scanners (per 100,000 persons) and the implementation rate of coronary computed tomography angiography (CCTA) for the 47 prefectures**


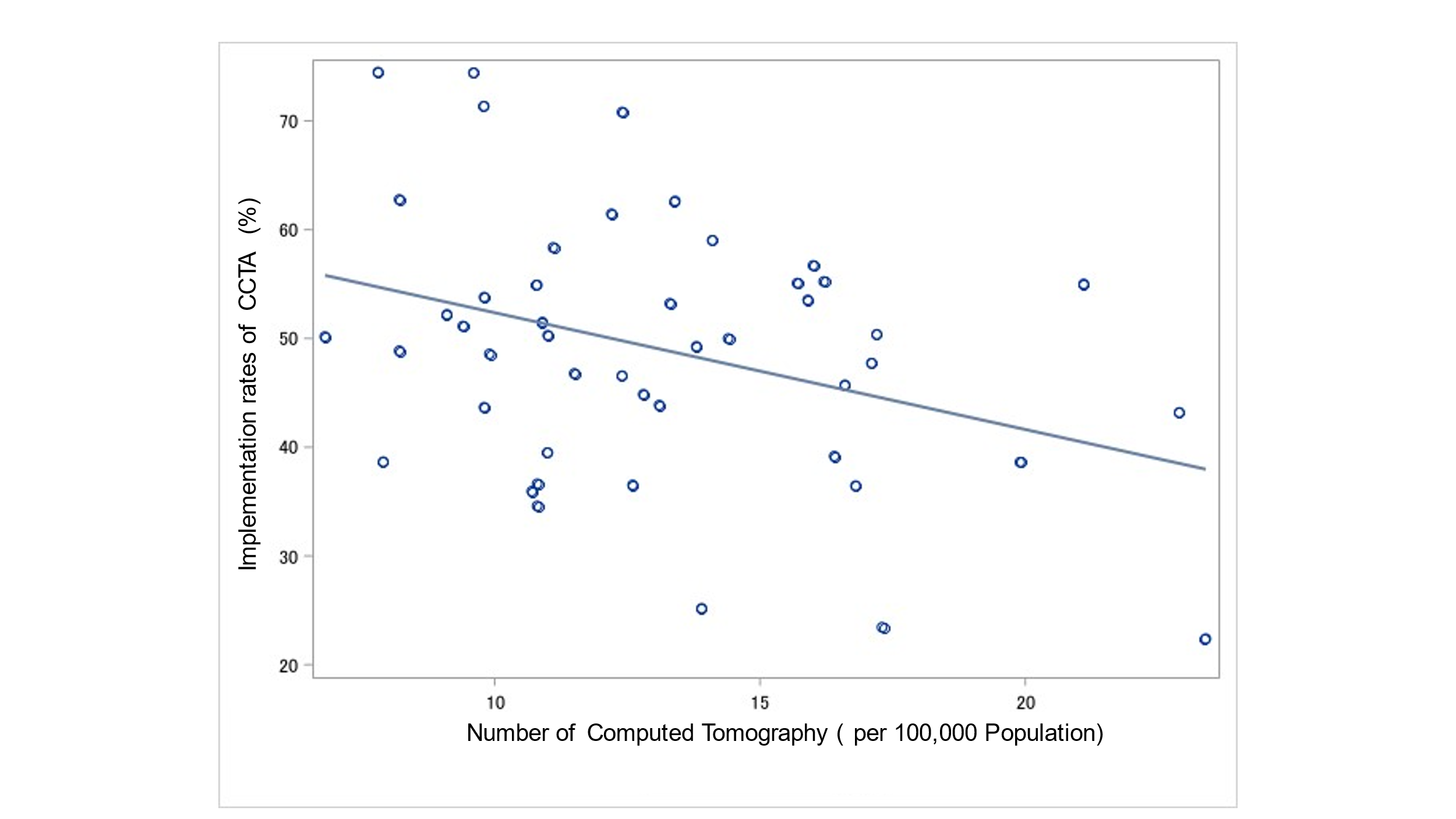

Supplement: Supplementary file 1 [file mmc1.docx]
